# Supplementary figures and images for: 3′HS1 CTCF binding site in human β-globin locus regulates fetal hemoglobin expression
Source: eLife. 2021 Sep 29;10:e70557. doi: 10.7554/eLife.70557 (PMC8500713; doi:10.7554/eLife.70557)

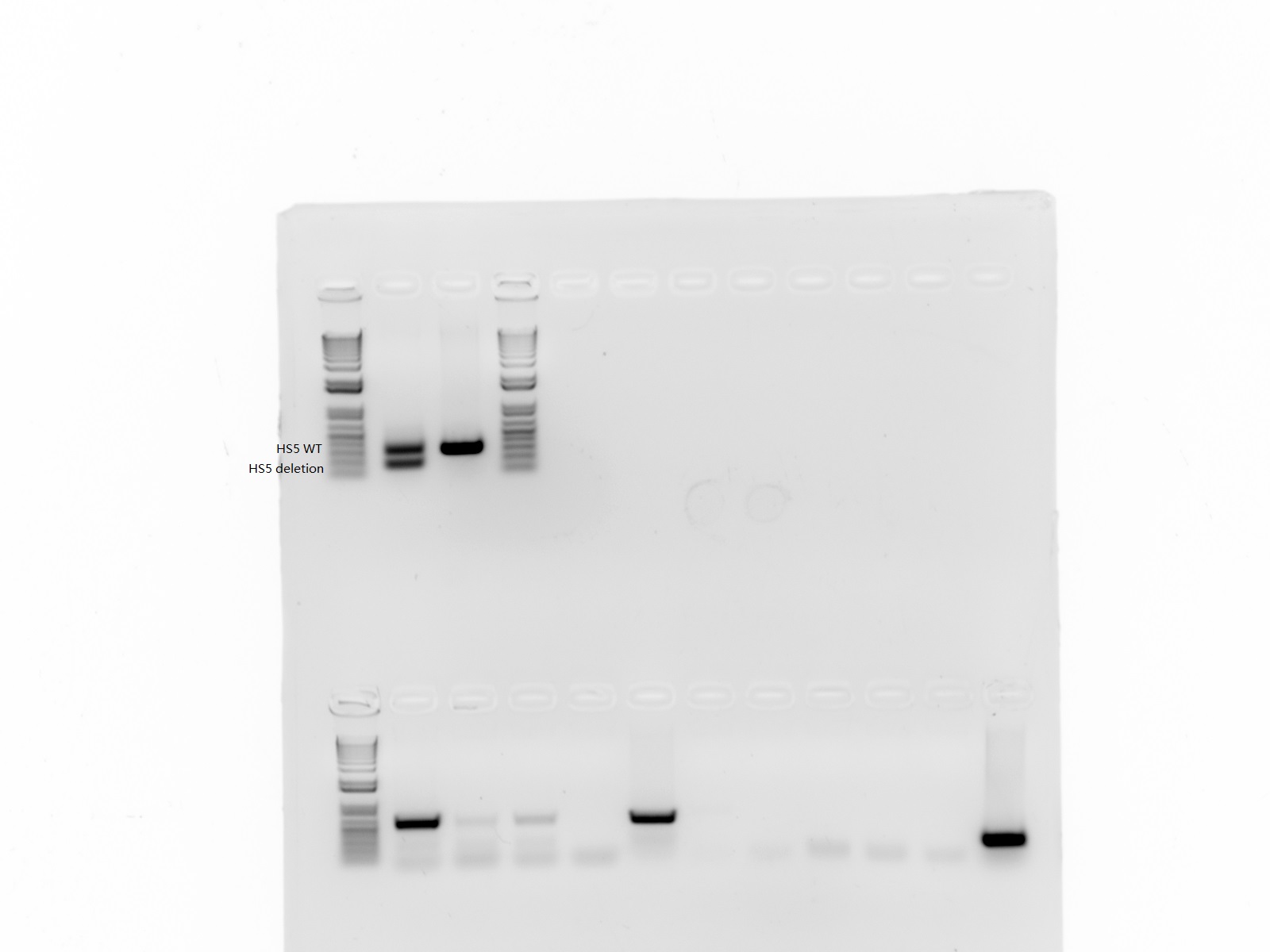

Supplement: Figure 1—figure supplement 1—source data 1. [file elife-70557-fig1-figsupp1-data1.zip › Figure 1_Figure Supplement1_source_data/16-5-18 hS5 del.jpg]

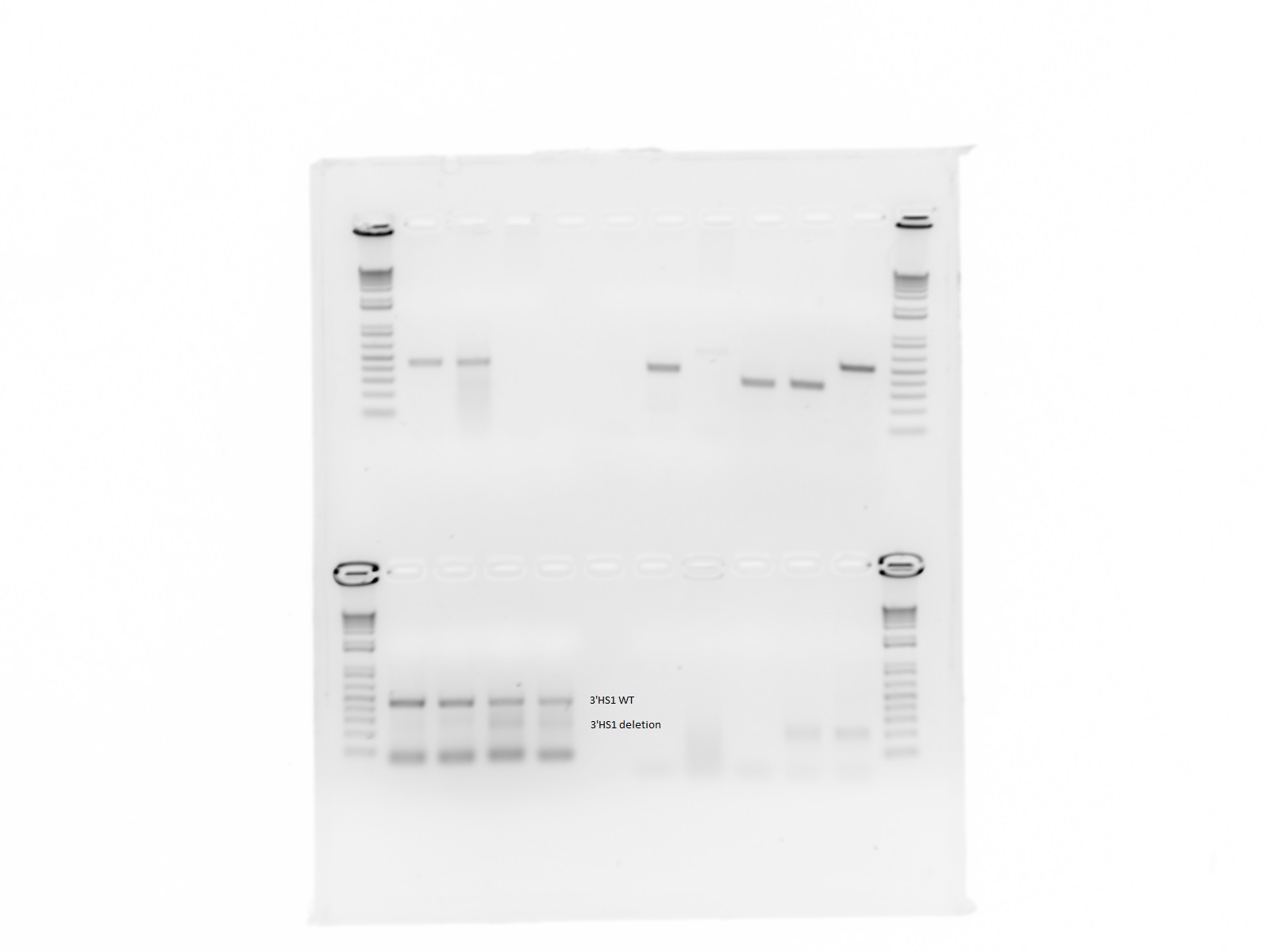

Supplement: Figure 1—figure supplement 1—source data 1. [file elife-70557-fig1-figsupp1-data1.zip › Figure 1_Figure Supplement1_source_data/16-5-18 h31 del.jpg]

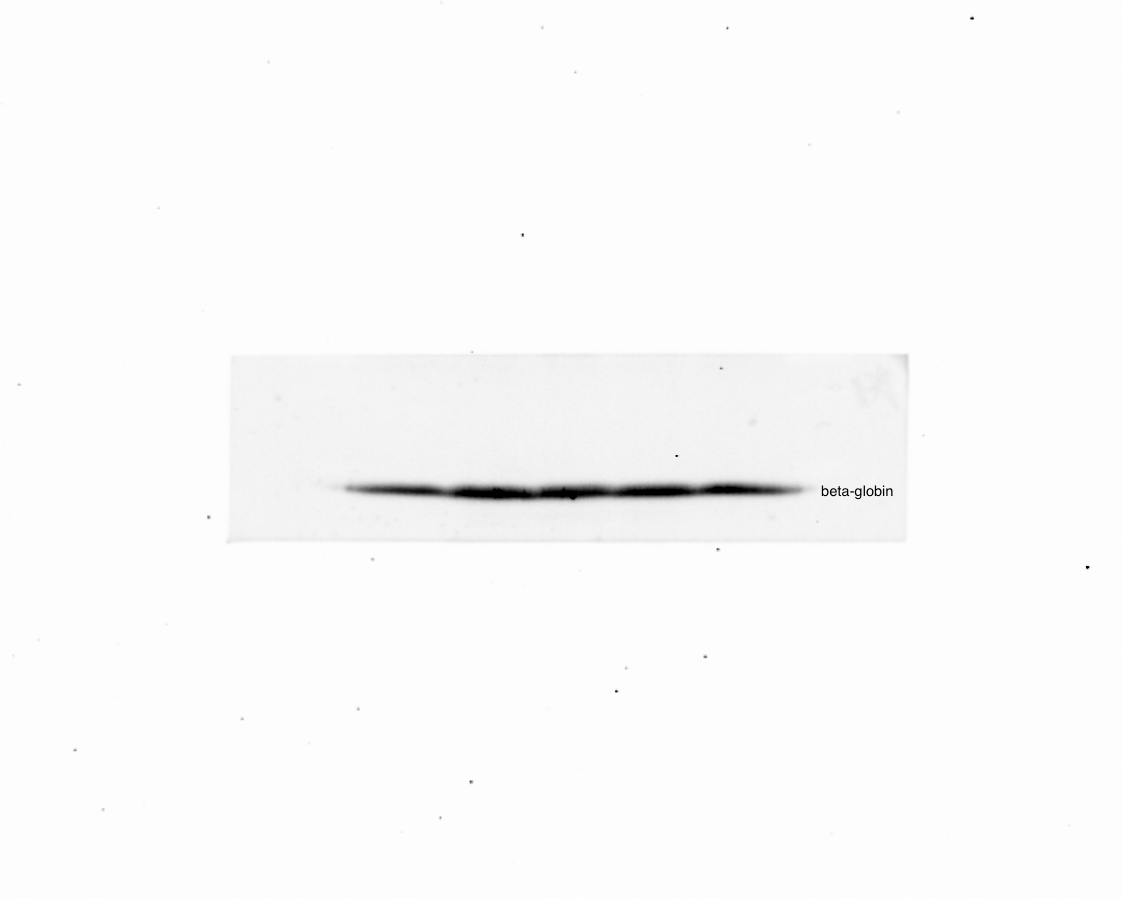

Supplement: Figure 2—source data 1. [file elife-70557-fig2-data1.zip › Figure 2_Source_data/2020-11-01 HBB, EDM3d2 (DyLight 800).tif]

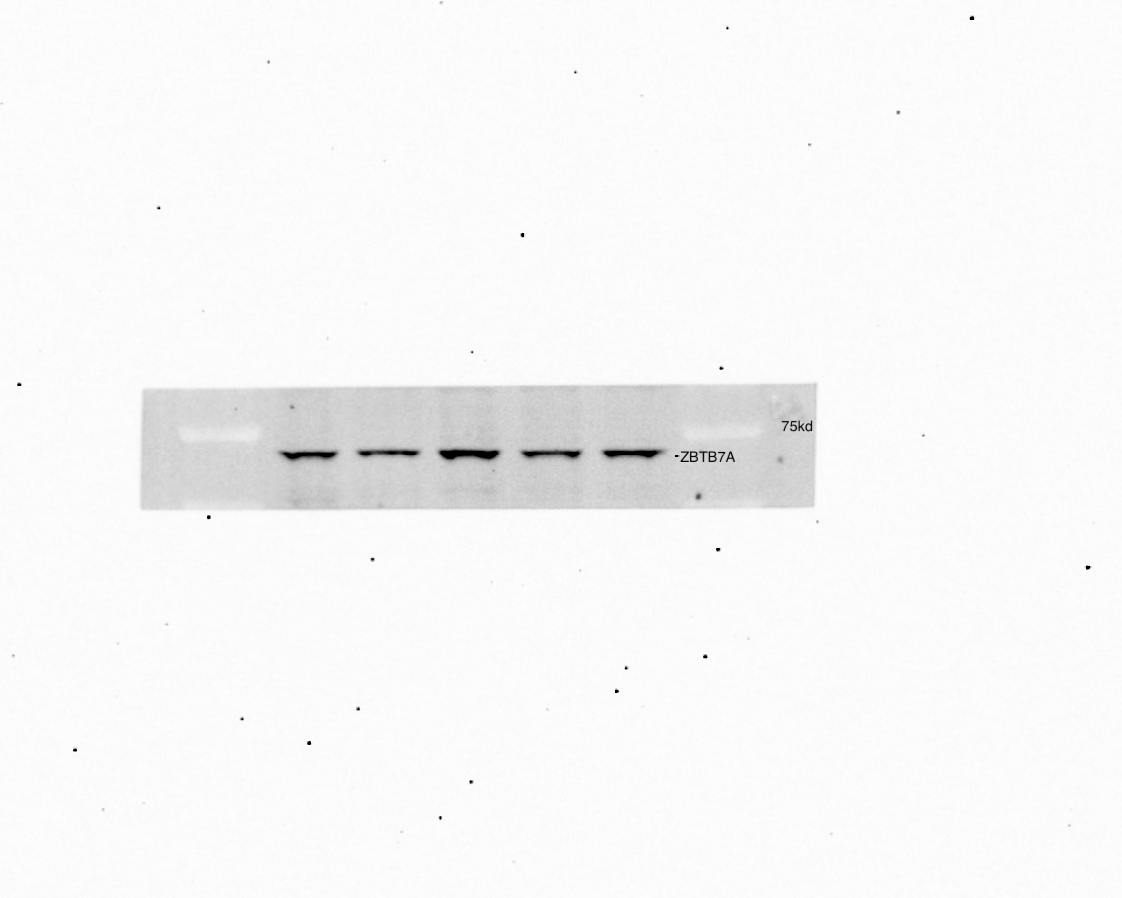

Supplement: Figure 2—source data 1. [file elife-70557-fig2-data1.zip › Figure 2_Source_data/2020-10-31 ZBTB7A, EDM3d2 (DyLight 800).tif]

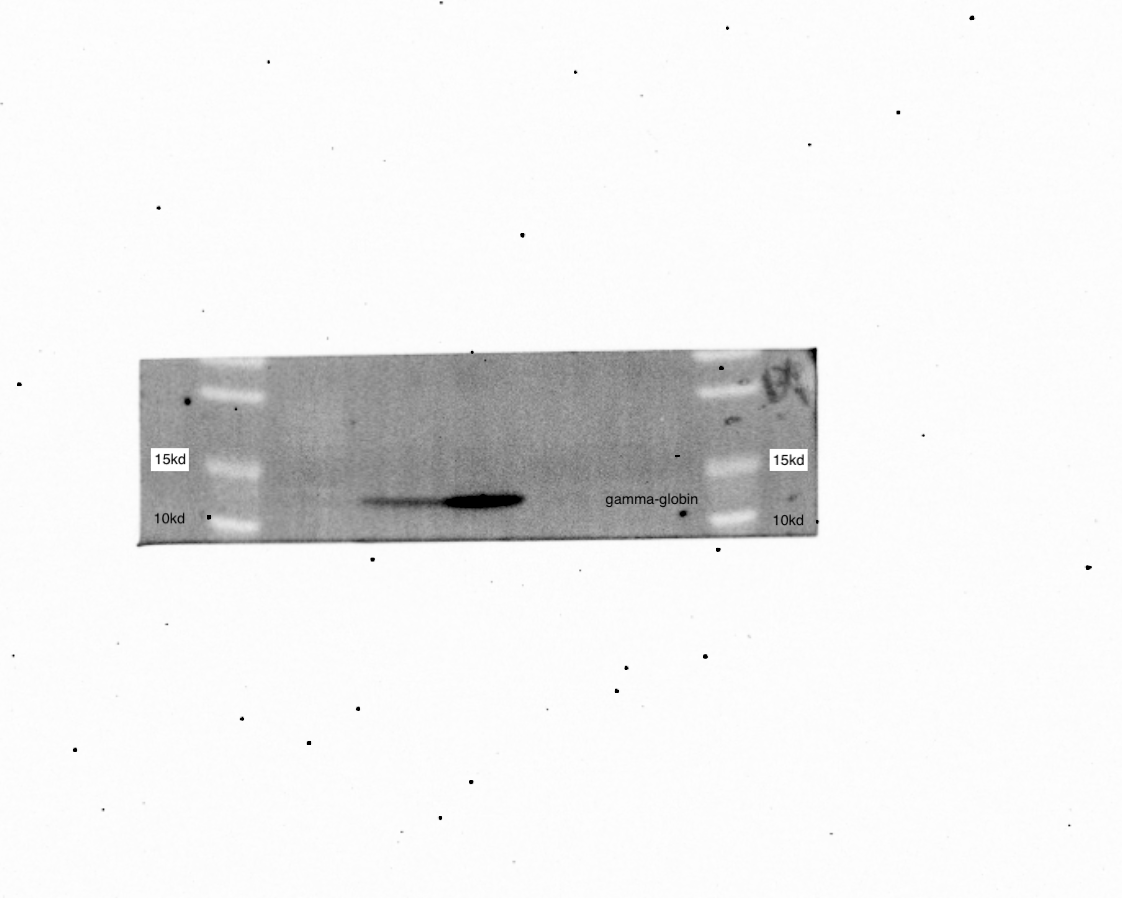

Supplement: Figure 2—source data 1. [file elife-70557-fig2-data1.zip › Figure 2_Source_data/2020-10-31 HbF, EDM3d2 (DyLight 800).tif]

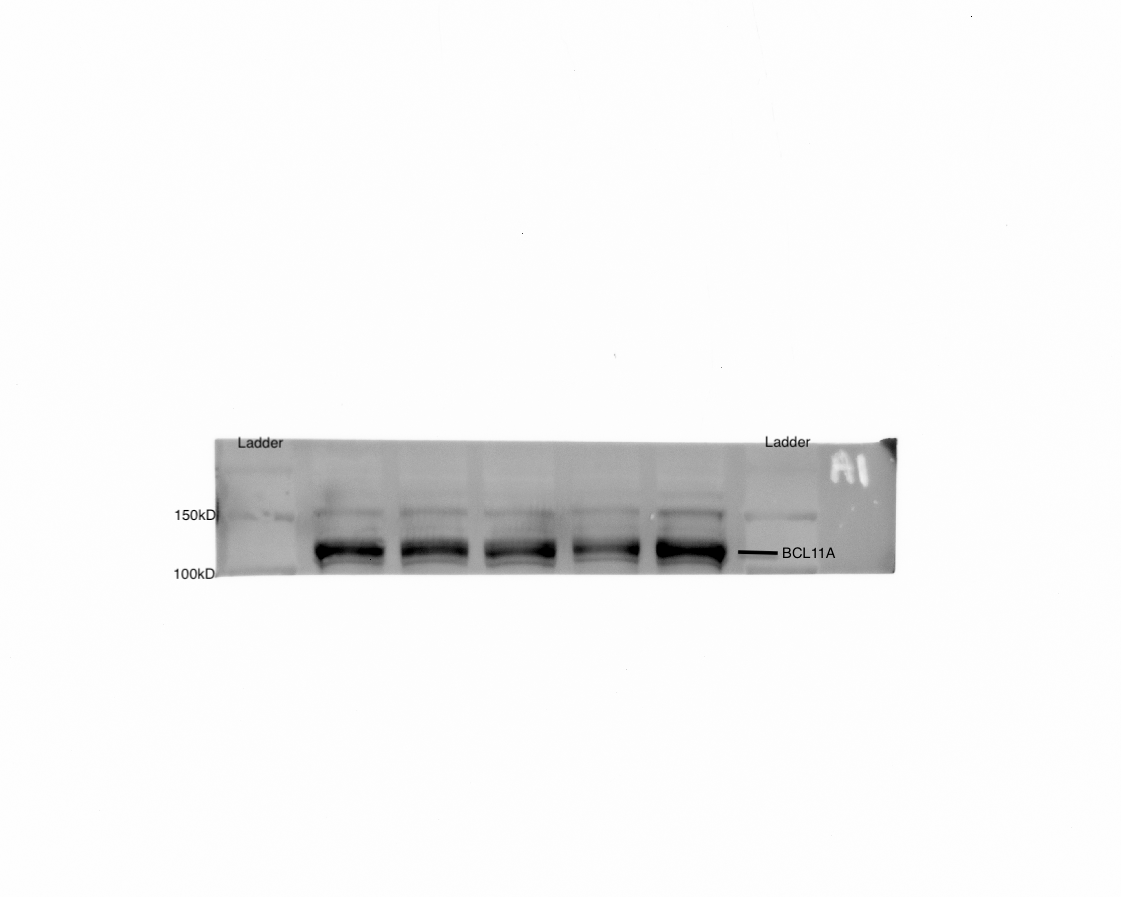

Supplement: Figure 2—source data 1. [file elife-70557-fig2-data1.zip › Figure 2_Source_data/2020-10-31 BCL11A, expansion medium(StarBright B700).tif]

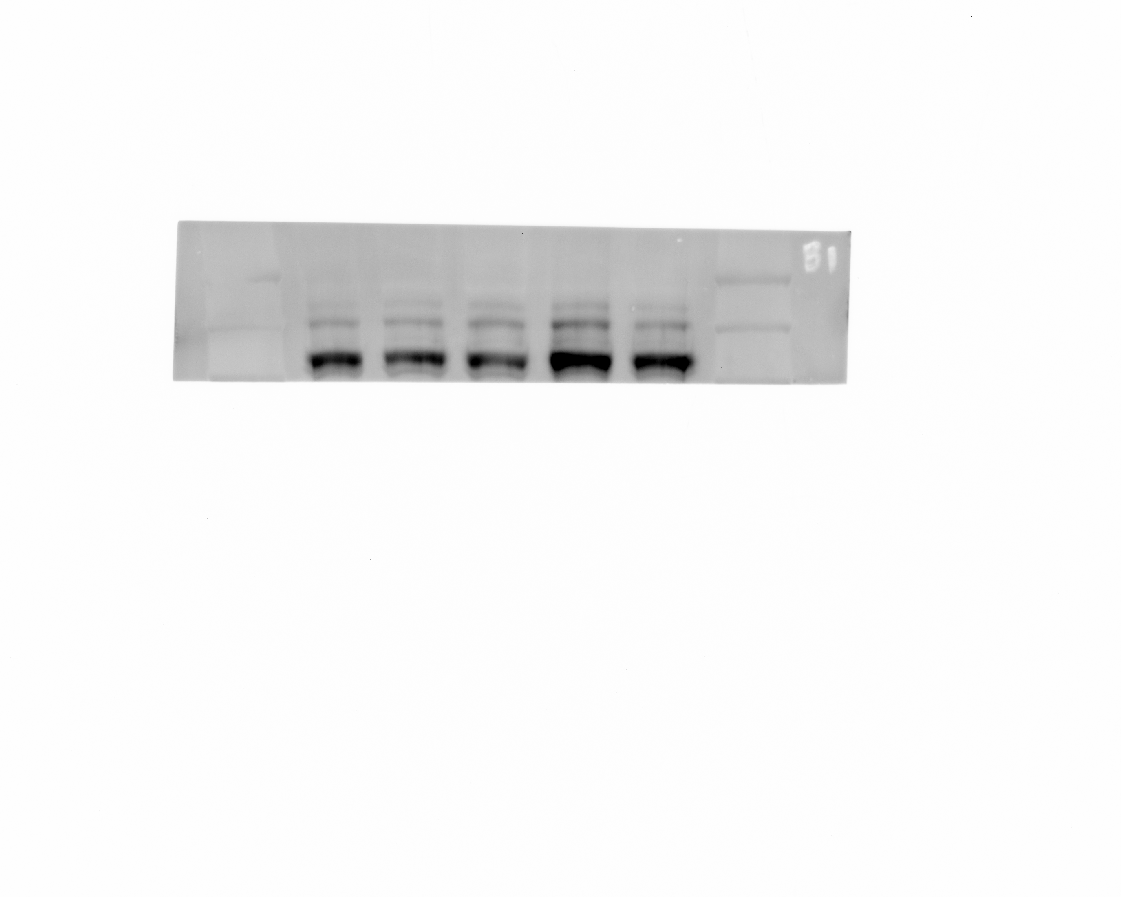

Supplement: Figure 2—source data 1. [file elife-70557-fig2-data1.zip › Figure 2_Source_data/2020-10-31 BCL11A, EDM3d2 (StarBright B700).tif]

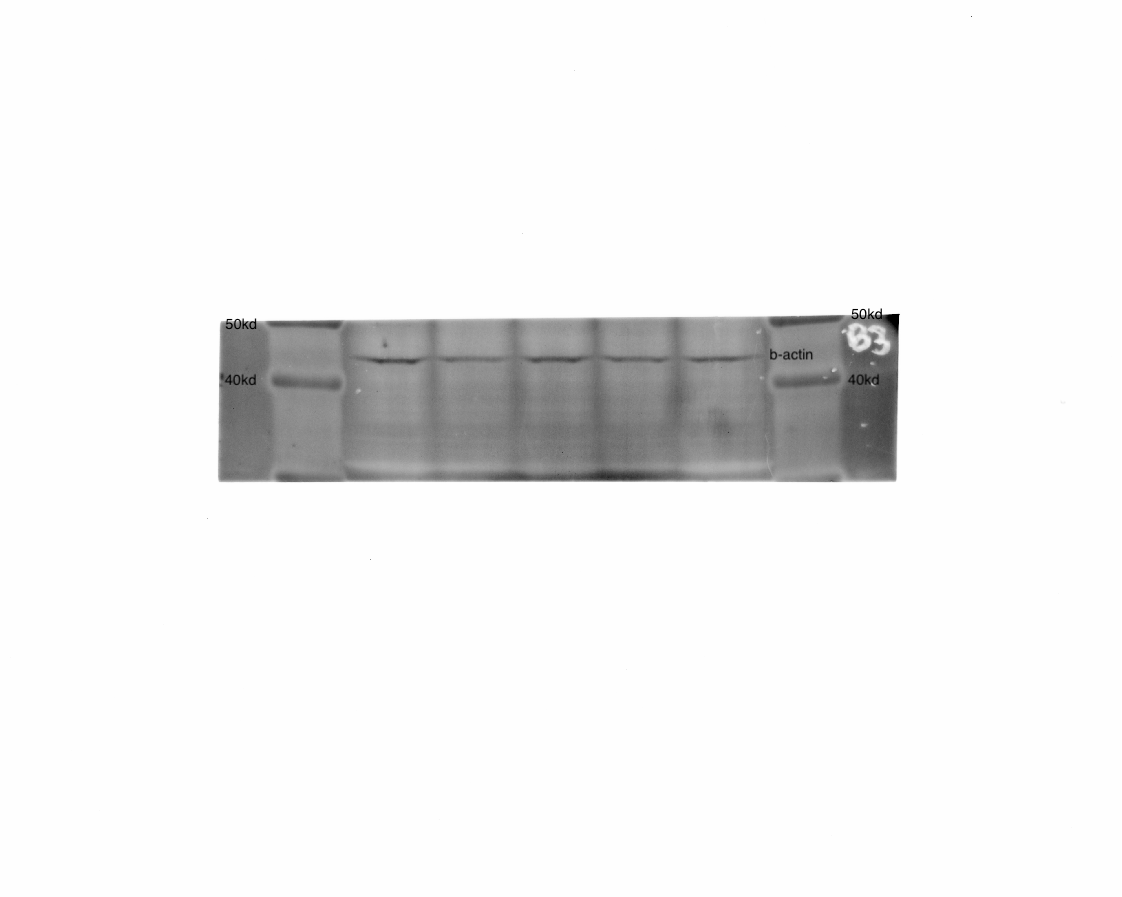

Supplement: Figure 2—source data 1. [file elife-70557-fig2-data1.zip › Figure 2_Source_data/2020-10-31 actinB, EDM3d2 (StarBright B700).tif]

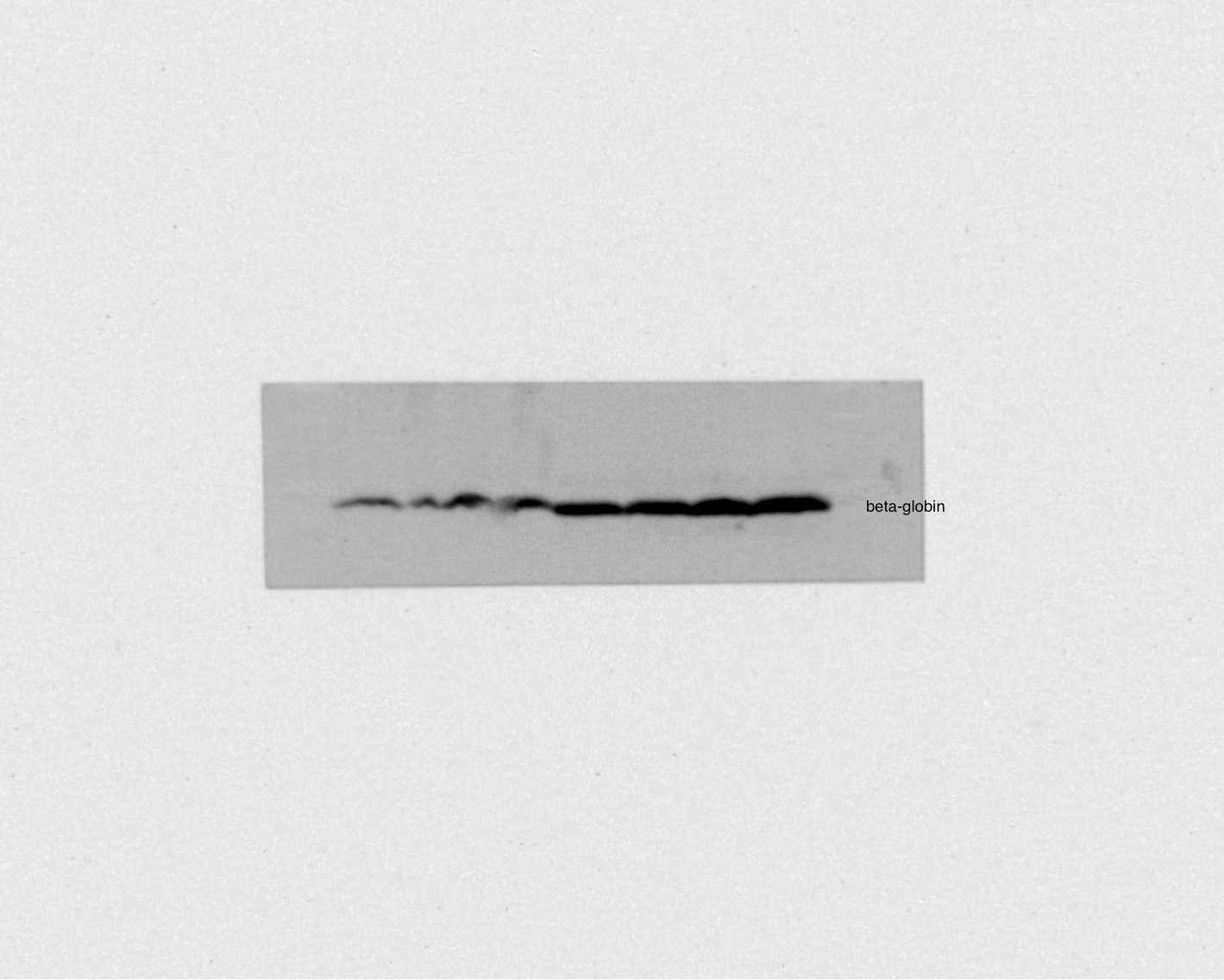

Supplement: Figure 2—figure supplement 1—source data 1. [file elife-70557-fig2-figsupp1-data1.zip › Figure2_Figure Supplement1_Source_data/2020-02-26 DyLight 800 89.865s(DyLight 800).tif]

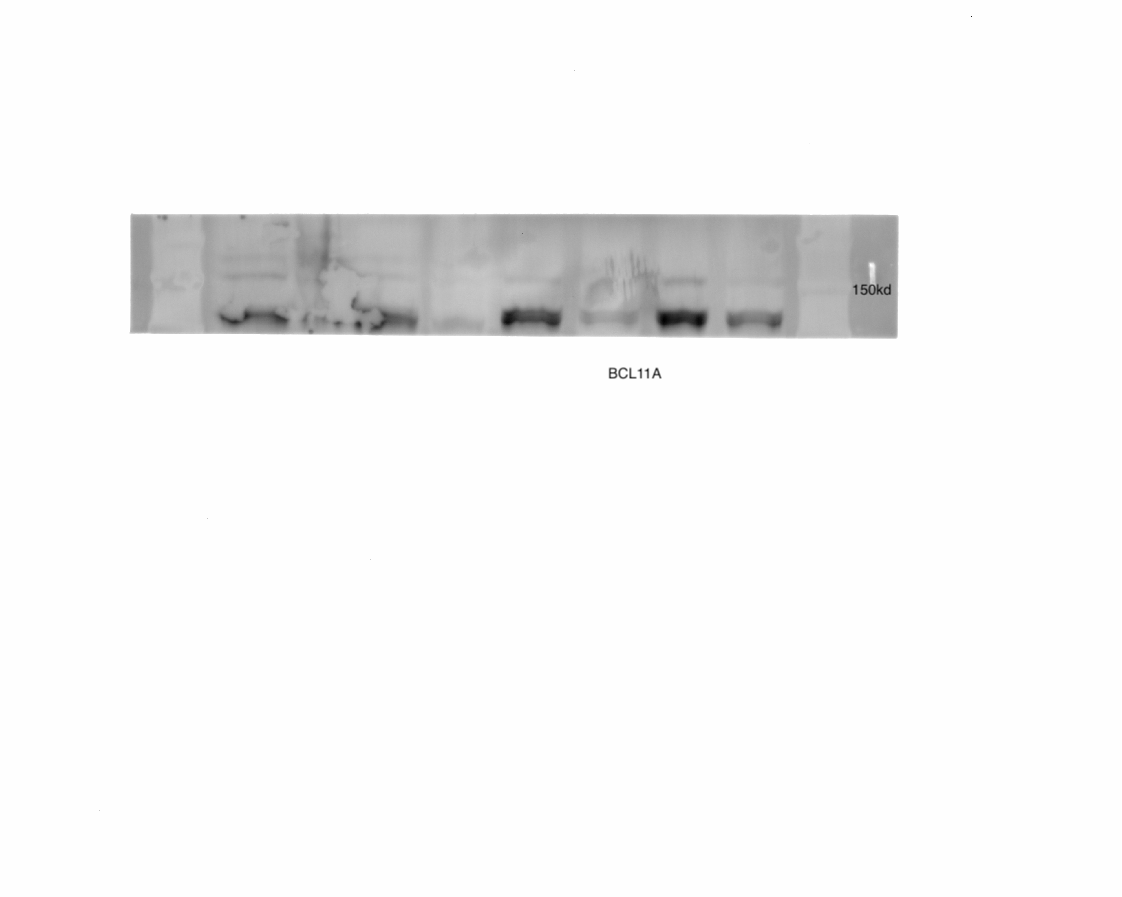

Supplement: Figure 2—figure supplement 1—source data 1. [file elife-70557-fig2-figsupp1-data1.zip › Figure2_Figure Supplement1_Source_data/2020-02-25 StarBright B700 62.100s(StarBright B700).tif]

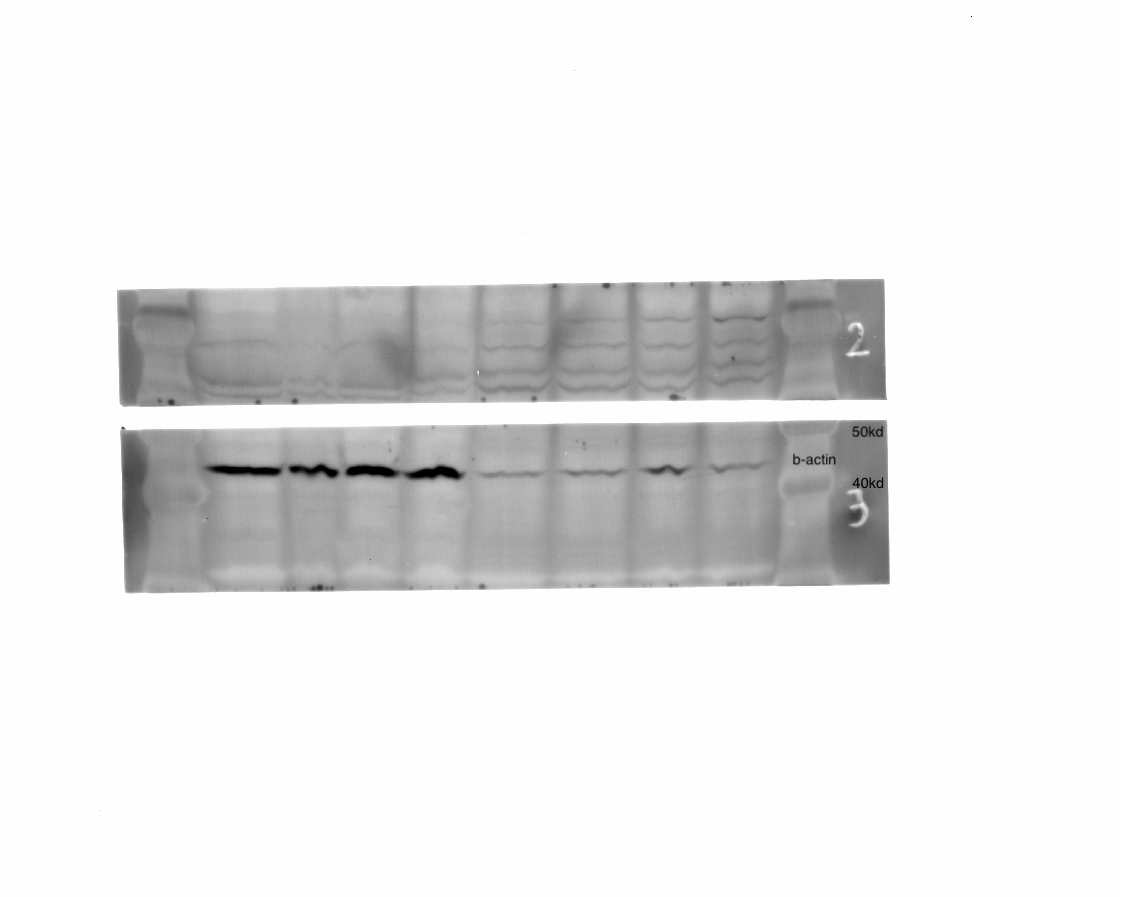

Supplement: Figure 2—figure supplement 1—source data 1. [file elife-70557-fig2-figsupp1-data1.zip › Figure2_Figure Supplement1_Source_data/2020-02-25 StarBright B700 61.753s(StarBright B700).tif]

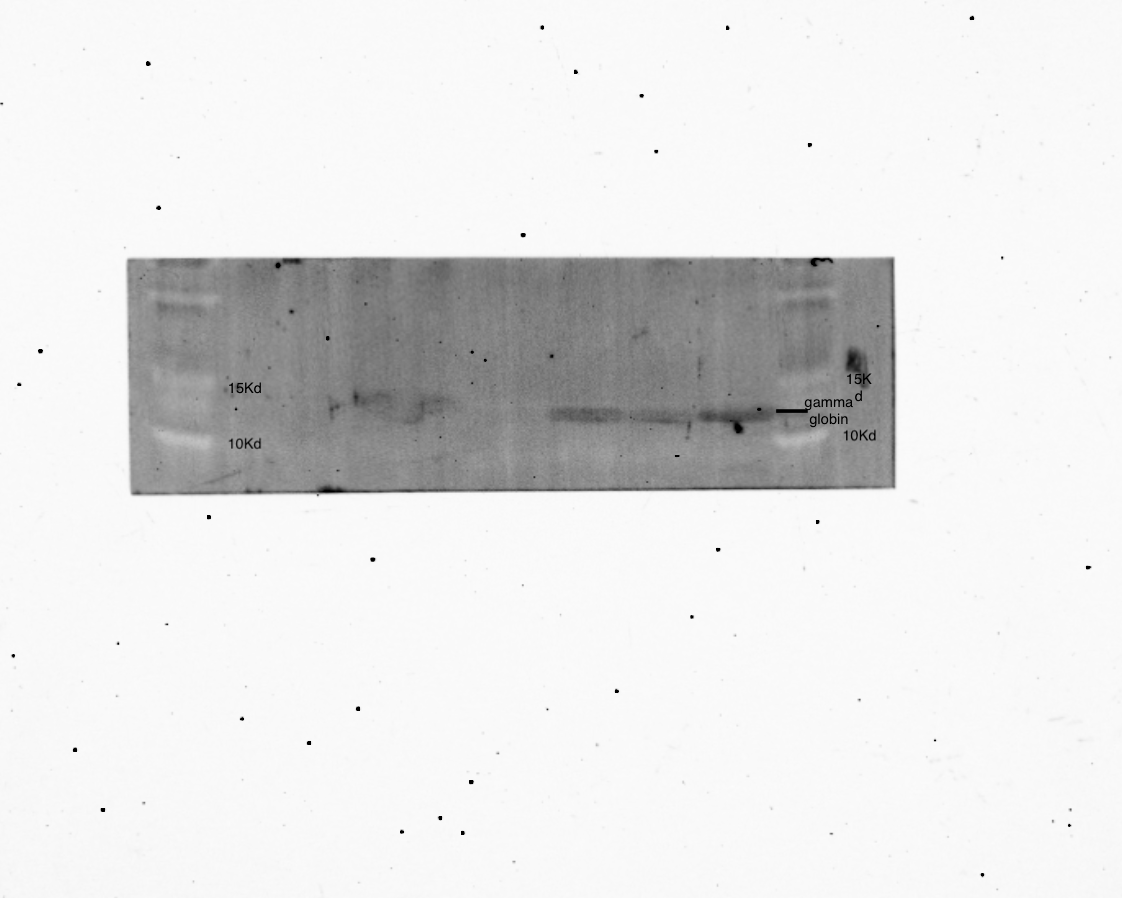

Supplement: Figure 2—figure supplement 1—source data 1. [file elife-70557-fig2-figsupp1-data1.zip › Figure2_Figure Supplement1_Source_data/2020-02-25 DyLight 800 300.000s(DyLight 800).tif]

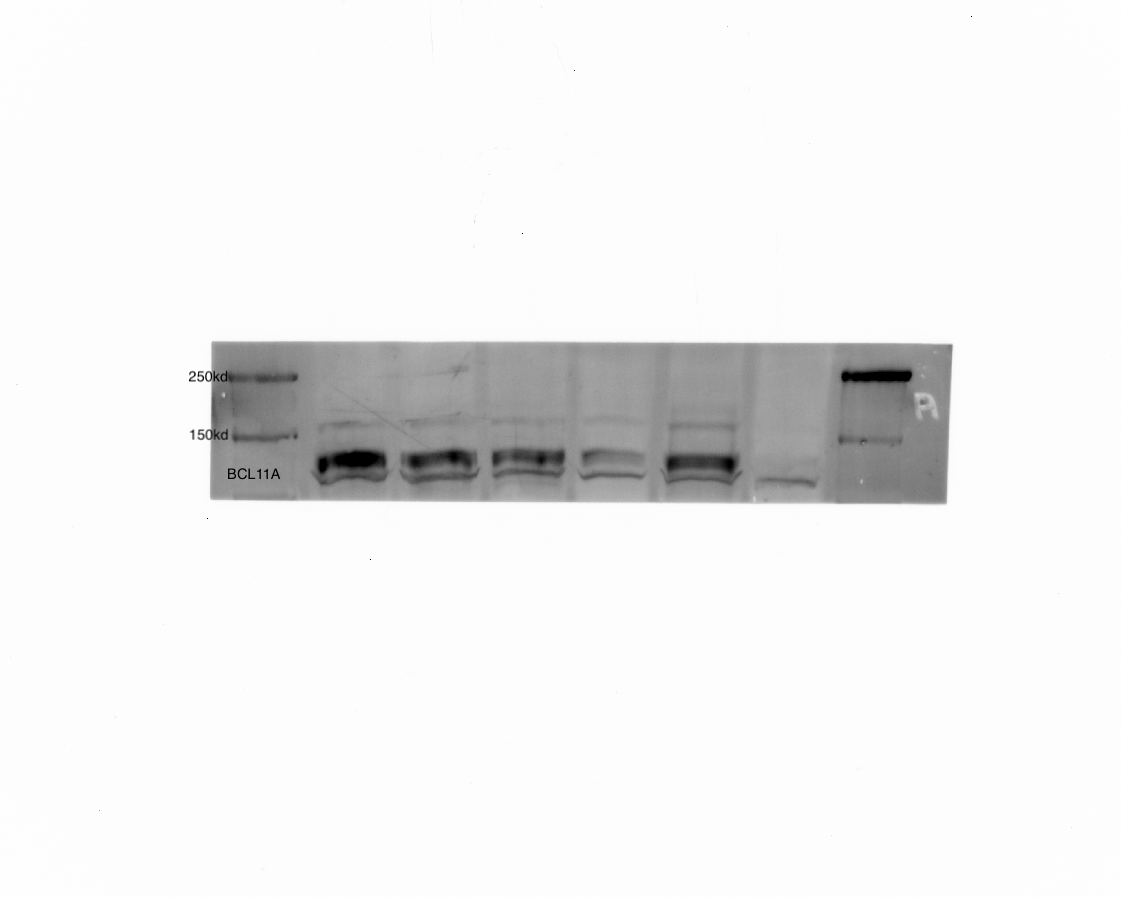

Supplement: Figure 2—figure supplement 2—source data 1. [file elife-70557-fig2-figsupp2-data1.zip › Figure2_Figure Supplement2_Source_data/2020-07-17 StarBright B700 60.000s(StarBright B700).tif]

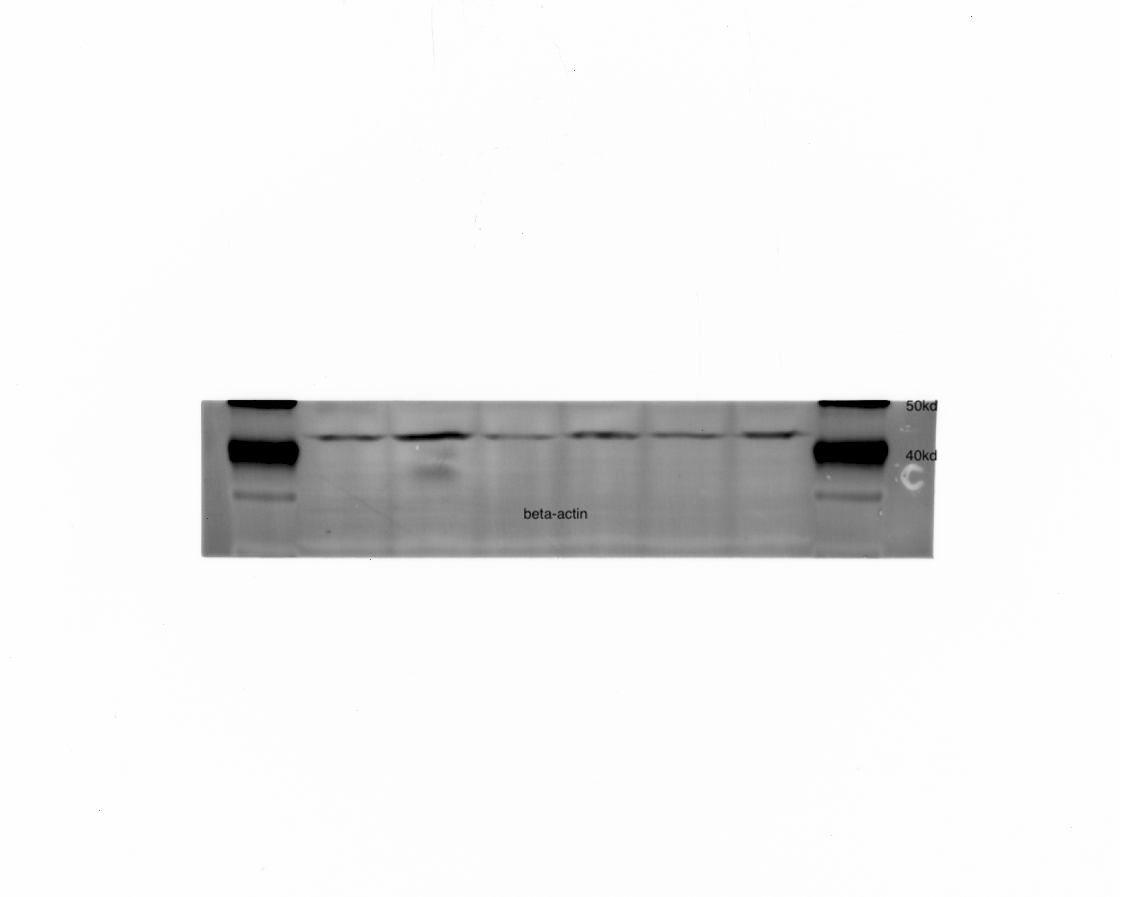

Supplement: Figure 2—figure supplement 2—source data 1. [file elife-70557-fig2-figsupp2-data1.zip › Figure2_Figure Supplement2_Source_data/2020-07-17 StarBright B700 240.000s(StarBright B700).tif]

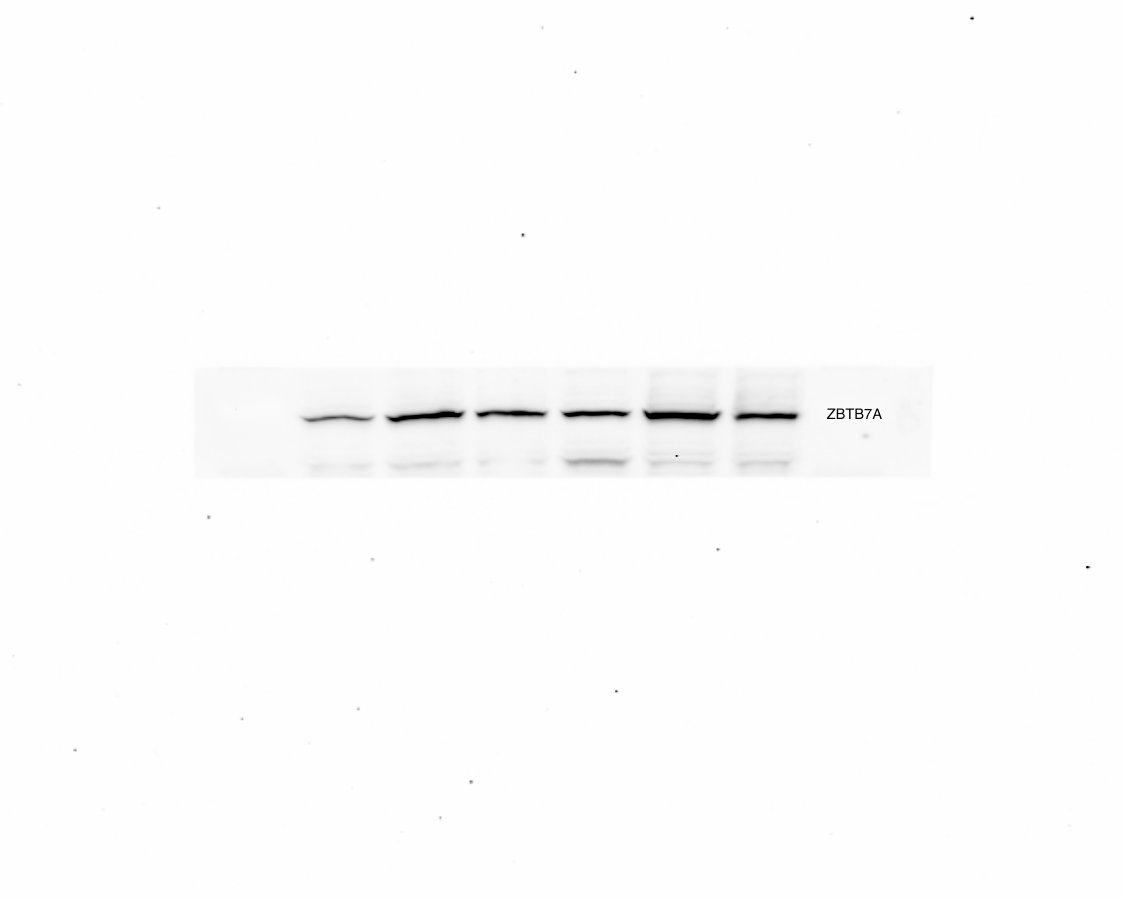

Supplement: Figure 2—figure supplement 2—source data 1. [file elife-70557-fig2-figsupp2-data1.zip › Figure2_Figure Supplement2_Source_data/2020-07-17 DyLight 800 60.000s(DyLight 800).tif]

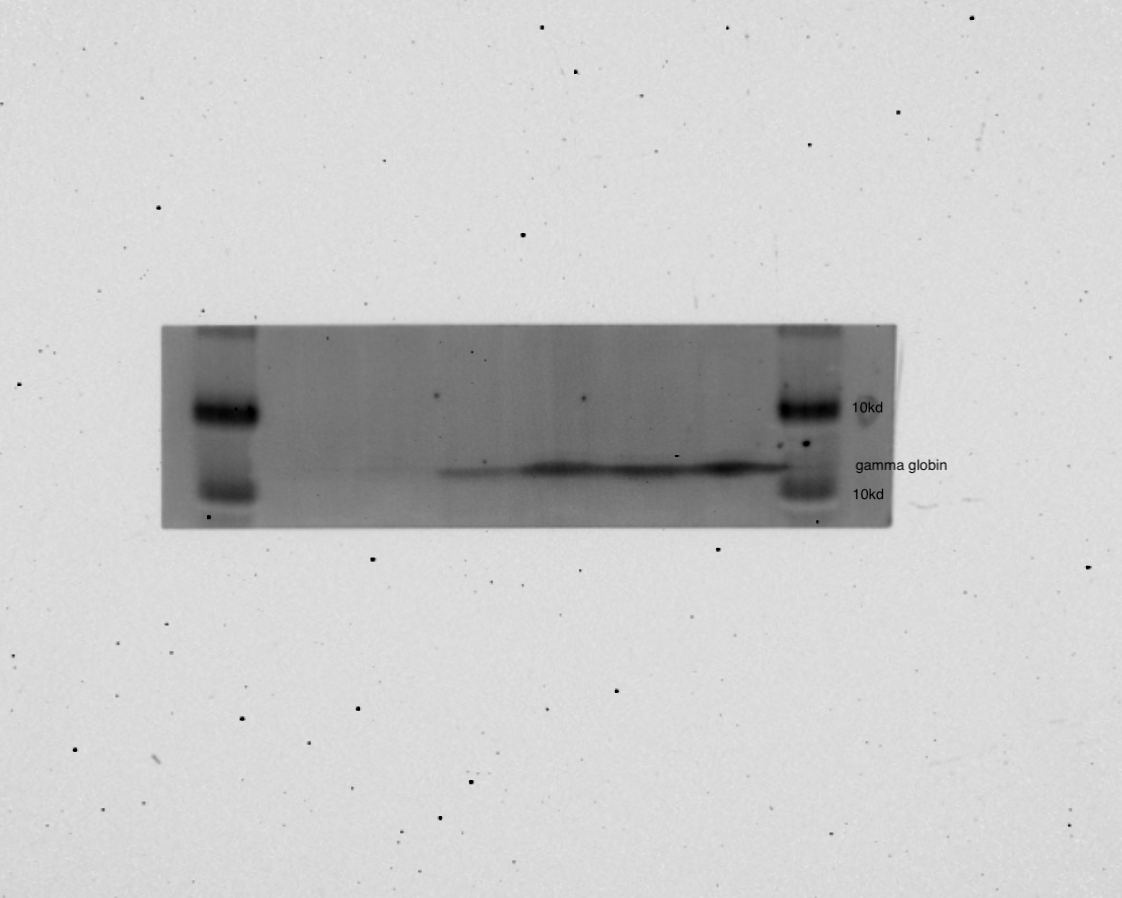

Supplement: Figure 2—figure supplement 2—source data 1. [file elife-70557-fig2-figsupp2-data1.zip › Figure2_Figure Supplement2_Source_data/2020-07-17 DyLight 800 180.000s(DyLight 800).tif]

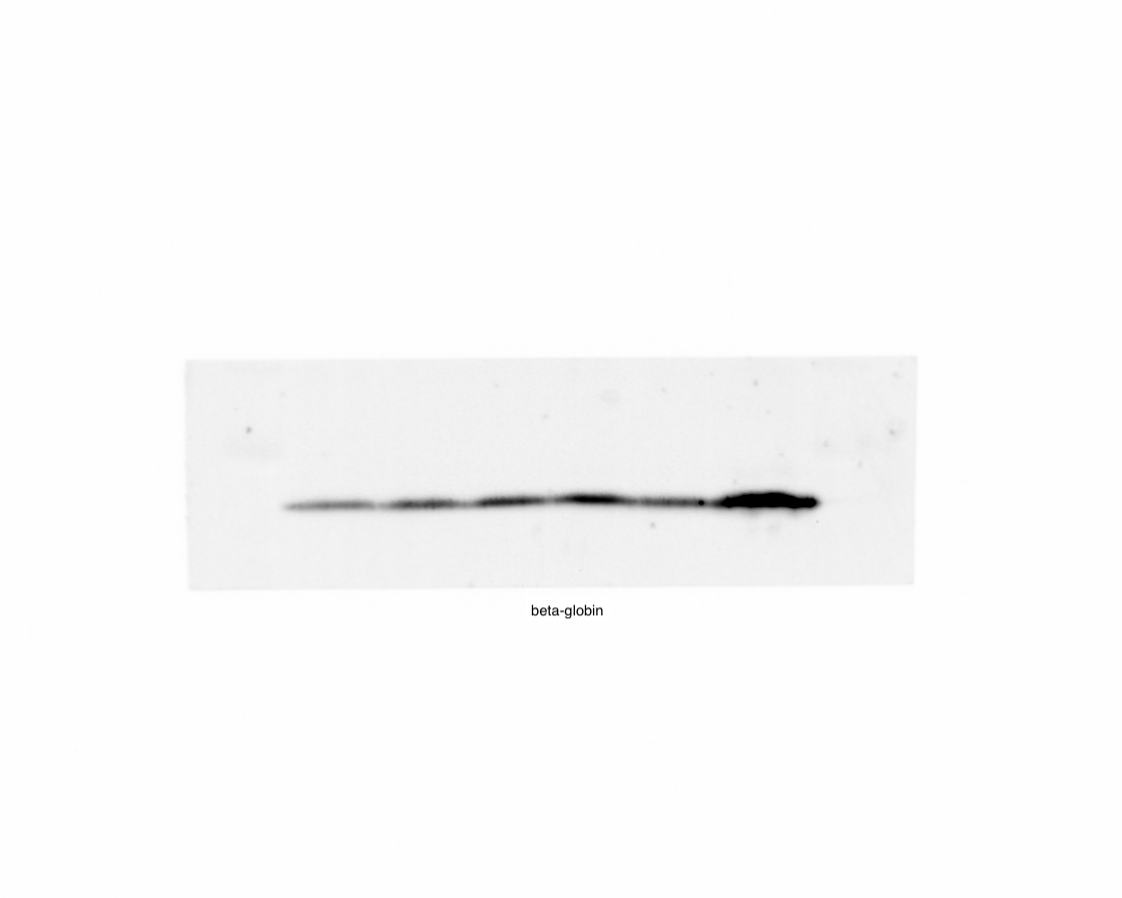

Supplement: Figure 2—figure supplement 2—source data 1. [file elife-70557-fig2-figsupp2-data1.zip › Figure2_Figure Supplement2_Source_data/2020-07-11 DyLight 800 49.367s(DyLight 800).tif]

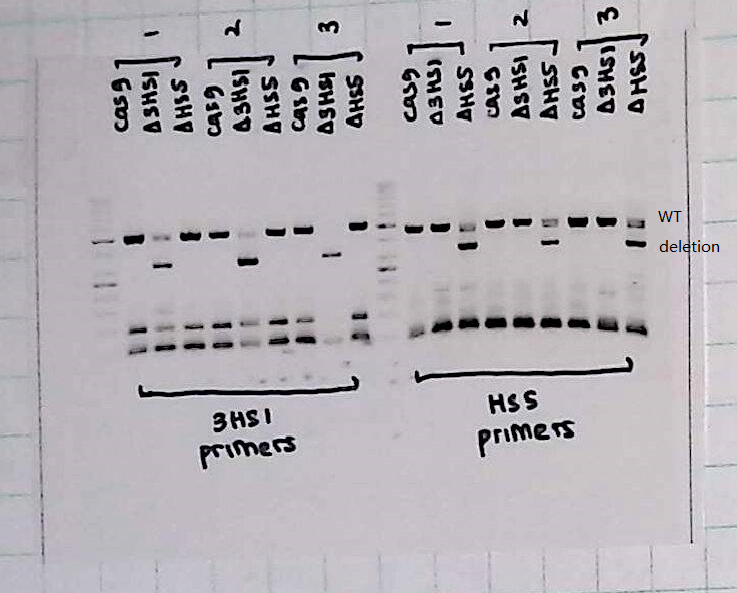

Supplement: Figure 4—source data 1. [file elife-70557-fig4-data1.zip › Figure 4_Source_data/Gel deletion-Figure 4.bmp]
